# Barley based gluten free beer – A blessing or an uncontrollable risk?

**DOI:** 10.1016/j.fct.2024.115019

**Published:** 2024-11

**Authors:** Elena Cubero-Leon, Charlotte B. Madsen, Katharina A. Scherf, Michelle L. Colgrave, Jørgen V. Nørgaard, Minna Anthoni, Katerina Rizou, Michael J. Walker, Ludvig M. Sollid

**Affiliations:** a European Commission, Joint Research Centre (JRC), Geel, Belgium; b National Food Institute, Technical University of Denmark, Lyngby, Denmark; c Leibniz Institute for Food Systems Biology at the Technical University of Munich, Freising, Germany; d Technical University of Munich, TUM School of Life Sciences, Professorship of Food Biopolymer Systems, Freising, Germany; e CSIRO Agriculture and Food, 306 Carmody Rd, St Lucia, QLD, 4067, Australia; f Finnish Food Authority, Mustialankatu 3, 00790, Helsinki, Finland; g General Chemical State Laboratory (GCSL), Athens, Greece; h Institute for Global Food Security, The Queen's University of Belfast, Belfast, BT9 5HN, Northern Ireland, UK; i Norwegian Coeliac Disease Research Centre, Institute of Clinical Medicine, University of Oslo, Norway and Department of Immunology, Oslo, University Hospital – Rikshospitalet, Oslo, Norway

**Keywords:** Gluten-free beer, Coeliac disease, Hydrolysis, Gluten quantification, Risk assessment, Reference materials

## Abstract

Recent reports have highlighted that beer labelled “gluten-free”, crafted with enzymatic treatments to remove gluten, may contain polypeptides that could be immunotoxic to individuals with coeliac disease. As strict adherence to a gluten-free diet is the only way to manage this condition, accurate labelling is crucial to those with coeliac disease. This paper aims to discuss the presence, levels and immunogenicity of gluten peptides found in gluten-reduced barley beers. While advances have been made in the detection and quantification of gluten peptides in beer, there are still challenges to the interpretation of gluten measurements as well as to assess whether peptides are immunotoxic in vivo. To make progress, future efforts should involve a combination of in vivo toxicity assessment of the degraded proteins, development of standardised gluten-free production strategies to minimise variability in gluten fragment presence, guidance on how to control the outcome as well as to develop appropriate reference materials and calibrators.

## Highlights

• Challenges remain to the interpretation of gluten measurements in beer.
• Future progress requires: in vivo toxicity assessment of the degraded proteins.
• Development of standardised gluten-free production strategies and control guidelines.
• Appropriate reference materials and calibrators based on the above findings.

## Graphical abstract

**Figure:**
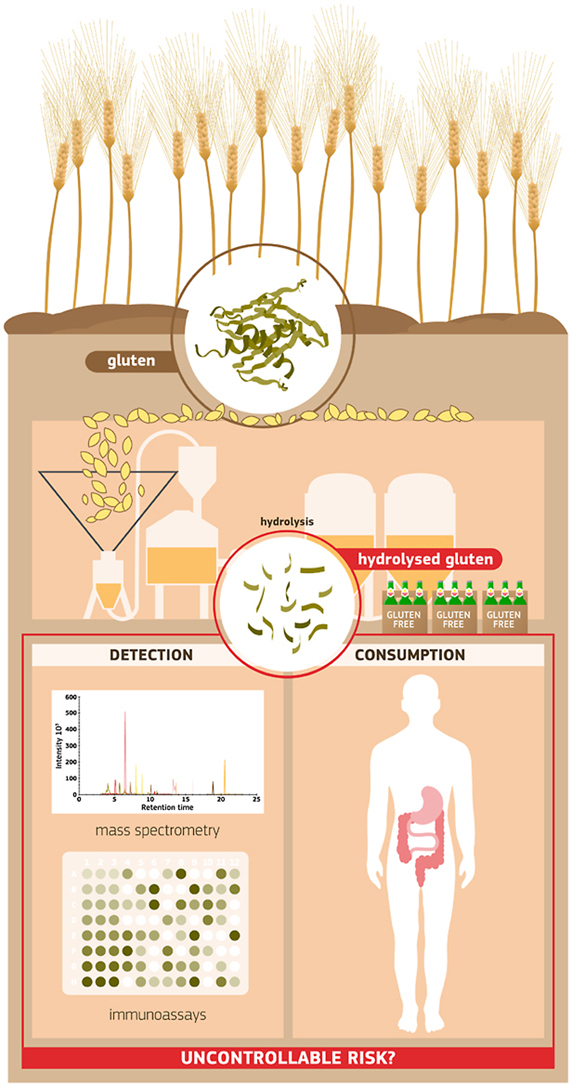

## 1 Introduction

Coeliac disease is a chronic immune-mediated condition caused by a maladapted immune response to gluten proteins in genetically susceptible individuals (Iversen and Sollid, 2023). The condition causes a lesion in the upper small intestine characterised by inflammation and tissue damage. Gluten proteins are storage proteins of wheat (gliadins and glutenins), rye (secalins), barley (hordeins) and crossbred varieties that generally are insoluble in water and physiological salt solutions. It is estimated that the overall worldwide prevalence of coeliac disease is about 1.4 % based on serologic tests and about 0.7 % based on small intestinal biopsy results (Singh et al., 2018). These estimates vary with geographical location, age and sex. The only way to manage the condition is by adhering to a strict life-long gluten-free diet. Therefore, labelling of food containing (or not containing) gluten plays an essential role in protecting subjects with coeliac disease from unwanted exposure to gluten.

Beer is the most consumed alcoholic beverage type in the European Region and the WHO Region of the Americas and the second most consumed in the world (World Health Organization, 2018). Beers crafted from non-gluten containing cereals are available on the market. These include beers made with sorghum, rice or millet (Hager et al., 2014). In contrast, traditional forms of beers are based on two gluten-containing grains like barley and, to a lesser extent, wheat, which makes them not suitable for consumption by people with coeliac disease. However, the gluten content in beer can be reduced by enzymatic proteolysis and filtration (Scherf et al., 2018; Wieser and Koehler, 2012). In the EU, these beers can be labelled as gluten-free if they contain gluten levels below 20 mg/kg of food as set in Commission Implementing Regulation (EU) No 828/2014, which cites Codex Alimentarius Commission, Standard for foods for special dietary use for persons intolerant to gluten, CXS 118–1979 (adopted in 1979, amended in 1983 and 2015 and revised in 2008). However, the reliability and accurate quantification of gluten in hydrolysed products such as beer, using current analytical methods is under debate (Scherf et al., 2021). As a result, the U.S. Food and Drug administration ruled that beer cannot be labelled “gluten-free” unless it is made from inherently gluten-free materials, like rice, (U.S. Food and Drug Administration, 2020). Brewers can instead label their beers as “gluten-reduced” or “crafted to remove gluten.” This ruling further requires the manufacturer must keep records to demonstrate the food is “gluten-free” before fermentation or hydrolysis, and free of contamination.

Recent reports employing various detection methods including mass spectrometry (MS) indicate that gluten-reduced beers may contain immunotoxic peptides, and this is so even for beers which are labelled as gluten-free following enzymatic treatments aiming to remove residual gluten peptides (Nye-Wood et al., 2023; Real et al., 2014; Spada et al., 2020). Similarly, residual material of gluten-removed (GR) barley-based beer was found to bind serum IgA or IgG of some patients with coeliac disease (Allred et al., 2017). Thus, it seems clear that many beers, even those that are considered gluten-free, can contain peptides which are potentially harmful to patients with coeliac disease. Whether these peptides would elicit immune reactions in vivo is however not clear, as such peptides may undergo rapid degradation when ingested. To the latter point, Spada et al. (2020) found that polypeptides from gluten-reduced beers able to induce an immune response in coeliac disease patients were drastically reduced after a simulated gastroduodenal digestion. The aim of the paper is to discuss the current evidence on the presence, levels and immunogenicity of gluten peptides in GR-barley beer. Current knowledge gaps and future needs for their safety assessment are highlighted.

## 2 Coeliac disease and immunotoxic gluten epitopes

**Fig. 1:**
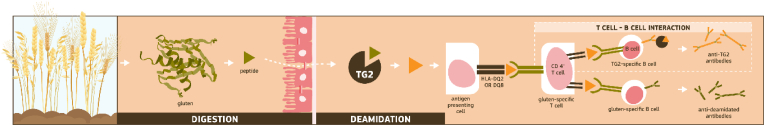
Diagram illustrating the immune response to gluten in coeliac disease.

Coeliac disease is a highly heritable disorder, and the main genetic component is the human leukocyte antigen (HLA), the major histocompatibility complex (MHC) in humans (Withoff et al., 2016). Certain HLA-DQ allotypes (DQ2.5, DQ2.2 and DQ8) are implicated in the pathogenesis by presenting gluten epitopes to CD4^+^ T cells (Sollid, 2017). Such gluten-specific CD4^+^ T cells drive the disease pathology (Iversen and Sollid, 2023). Once primed, the gluten-specific CD4^+^ T cells persist in coeliacs for decades thus explaining why coeliac disease is a life-long condition (Risnes et al., 2018). While HLA-DQ2.5, HLA-DQ2.2 and HLA-DQ8 differ in their binding specificity and thereby present to T cells unique sets of peptides, they all prefer binding of peptides with negatively charged amino acids (Tollefsen et al., 2006). Gluten proteins in general have few negatively charged residues. However, in coeliac patients modification of gluten via deamidation of glutamine to glutamate mediated by transglutaminase 2 (TG2) permits T-cell responses (Fig. 1). (Molberg et al., 1998; van de Wal et al., 1998). The enzyme typically targets glutamine residues in the sequence motif QXP(F) (Fleckenstein et al., 2002; Vader et al., 2002). TG2 is also an autoantigen in coeliac disease. The patients generate highly disease-specific antibodies to this enzyme when they consume gluten (Dieterich et al., 1997). This dual role of TG2 in coeliac disease pathogenesis by both being a target for autoantibodies and by being critical for making gluten immunogenic is hardly coincidental. Likely, the two functions are interrelated. This can happen by B cells serving as antigen presenting cells to T cells (Fig. 1). Conceivably, B cells specific for TG2 can present deamidated gluten peptides to CD4^+^ gluten-specific T cells by involvement of hapten-carrier like TG2-gluten complexes (Sollid et al., 1997). On binding and internalization of TG2-gluten complexes by TG2-specific B cells, deamidated gluten peptides are generated in endosomes. Such deamidated peptides can bind to HLA-DQ molecules for subsequent presentation to CD4^+^ gluten-specific T cells when reaching the cell surface of the B cells (Fig. 1). The gluten epitopes that stimulate CD4^+^ T cells of coeliacs typically reside in the parts of gluten which are rich in proline residues. This is so because proline is part of the sequence motifs that are targeted by TG2 (Fleckenstein et al., 2002; Vader et al., 2002) and because proline makes peptides resistant to gastrointestinal digestion (Shan et al., 2002) thus better preserving peptides that can be recognised by CD4^+^ T cells (i.e., longer than 10–12 amino acids). Further, the proteolytic resistance of long gluten peptides fragments may be augmented by the ability of such peptides to self-assemble into oligomers (Herrera and Dodero, 2021).

It follows that the main gluten peptides which people with coeliac disease should avoid are those which become targeted by TG2, and which are presented by the HLA-DQ2.5, HLA-DQ2.2 or HLA-DQ8 molecules to gluten-specific CD4^+^ T cells (Sollid, 2002). As different gluten peptides are presented by HLA-DQ2.5, HLA-DQ2.2 or HLA-DQ8, the lists of coeliac immunotoxic gluten epitopes are unique for each of the three HLA-DQ allotypes (Sollid et al., 2012). Using data from 16 publications, a compilation of 38 T-cell epitopes from wheat, rye, barley, oats were listed (Sollid et al., 2020). Among these 38 T-cell epitopes, there are 28 unique sequences to wheat as some sequences are present in more than one cereal or as some sequences are presented by more than one HLA-DQ allotype. The epitopes are represented in this list as the nine-mer core regions which interact with the HLA-DQ molecules. The peptides are generally only stimulatory for T cells in their deamidated form, yet also native version of the peptides would represent immunotoxic sequences when consumed in food as the deamidation would happen in vivo. The epitopes are named with information about HLA presenting element involved and the proteins harbouring the sequence. To give some examples, DQ2.5-glia-ω1 and DQ2-5-hor1 are two DQ2.5 restricted T-cell epitopes with the native version of the sequences found in ω-gliadins and hordeins, respectively while DQ2.2-glut-L1 is a DQ2.2-restricted T-cell epitope with the native version of the sequence found in low-molecular weight glutenins and DQ8-glia-α1 represents a T-cell epitope with the native version of the sequence found in gliadins. This 2020 publication is an update of a previous report by Sollid et al. (2012), which detailed the criteria required for a sequence to be defined as a T-cell epitope. These criteria were: “1: Reactivity against the epitope must have been defined by at least one specific T-cell clone; 2: The HLA-restriction element involved (chiefly DQ2.5, DQ2.2 or DQ8) must have been unequivocally defined and; 3: The nine-amino acid core of the epitope must have been defined either by an analysis with truncated peptides and/or HLA-binding with lysine scan of the epitope or comparable approach”. The T-cell receptor of a CD4^+^ T cell generally interacts with residues on each side of the nine-mer core region. Thus, two peptides which share an identical nine-mer core region, but which differ in their sequence outside of the core often differ in their ability to stimulate T cells. Expanding the sequence search beyond nine-amino acids thus would lead to a longer list of immunotoxic epitopes.

Patients with coeliac disease have serum IgA and IgG antibodies to deamidated gluten peptides (Osman et al., 2000). This antibody response is strikingly focused, and the sequence QPEQPFP is preferentially targeted (Osman et al., 2000; Zhou et al., 2022). Whether a sequence targeted by coeliac antibodies alone should be defined as an immunotoxic sequence by this feature is not obvious. Of note, however, such antibody epitopes typically locate spatially close to T-cell epitopes within the gluten proteins. For instance, the QPEQPFP sequence is overlapping with or adjacent to several T-cell epitopes of ω-gliadins (DQ2.5-glia-ω1 and DQ2.5-glia-ω2) and γ-gliadins (DQ2.5-glia-γ4c and DQ2.5-glia-γ5) (Dørum et al., 2016).

While this list of nine-mer gluten T-cell epitopes is extensive, synthetic peptides representing this list can only account for the reactivity of 50% of gluten-specific T cells of coeliac disease patients implicating that many epitopes are yet to be defined (Sollid et al., 2020). While epitopes of wheat are best characterised, recent results suggest that most of the yet unidentified epitopes will be shared between wheat, barley and rye (Chlubnová et al., 2023).

In addressing whether a food item will be dangerous to coeliac disease patients, the key issue to consider is whether the food item containing gluten will give rise to peptides that reach the small intestinal mucosa and stimulate CD4^+^ T cells. The fact that gastrointestinal digestion of free peptides in liquids (like those in beer) is likely to be different from the one of intact proteins within a solid matrix, complicates their immunotoxicity assessment.

## 3 Gluten legal limits

Most legal limits for gluten in gluten free products derive from Codex Alimentarius standards in 2005, which initially proposed a gluten limit of 200 mg gluten/kg of food. However, in the Codex standard CXS 118–1979 revised in 2008 the limit was reduced to 20 mg of gluten/kg of food (Codex Alimentarius, 2008). It had been evident for many years that the 200 mg of gluten/kg of food limit was not acceptable (EFSA, 2004). The challenge was to have both a scientific basis for the establishment of gluten levels and a method for determination of the gluten levels (Codex Alimentarius Commission, 2005). Based on the Codex reports, it can be inferred that the main factor behind the 20 mg of gluten/kg of food limit set in the standard was the protection of people with coeliac disease, but with an important contributory factor being the capacity to measure the gluten content of foods. The critical clinical study used appears to have been the double-blind placebo-controlled challenge study by Catassi et al. (2007). In this study adults with coeliac disease, belonging to three groups with thirteen participants each, were exposed daily for 90 days to either placebo, 10 or 50 mg gluten in a blinded manner. Participants were asked to maintain a strict gluten free diet during the study and were assumed to consume less than 5 mg gluten/day from their diet. The response to gluten was measured as change in small intestinal morphology before and after challenge. Based on statistical differences the authors concluded that ingestion of gluten should be kept lower than 50 mg/day for treatment of coeliac disease, but also stated that “the gluten micro challenge disclosed large interpatient variability in the sensitivity to gluten traces. Some patients showed a clear-cut worsening of the small-intestinal architecture after ingesting only 10 mg gluten/day, whereas others had an apparent improvement in mucosal histology after the 3-month challenge with 50 mg gluten/day.” At not more than 20 mg of gluten/kg of food described as gluten-free, and an estimated daily intake of 500 g of gluten-free food, a coeliac patient is unlikely to consume more than 10 mg gluten a day. Therefore, the limit of detection of 10 mg of gluten/kg for the analytical method referred to in the Standard CXS 118–1979 as a type 1 method for gluten analysis, the Mendez R5 ELISA, means a ‘not detected’ result for gluten is less likely to give rise to an intake of more than 5 mg of gluten per day. It should be noted that the Codex Alimentarius Commission, as well as other contributing parties, understood that 20 mg of gluten/kg of food (or rather the possible resulting maximum intake) was a maximum tolerable daily intake rather than a No Observed Adverse Effect Level (NOAEL).

The development of probabilistic risk assessment methods for food allergy recently has allowed better use of challenge data. This knowledge was used in a study by Rasmussen et al. (2022) using data from Catassi et al. (2007) to form a distribution of the doses causing changes in small intestinal morphology. In this way, all data are used and as a result the probabilistic risk assessment predicts a daily gluten intake around 10 mg as an adverse event in a proportion of persons with coeliac disease.

In addition to the 20 mg of gluten/kg of food level, Commission Implementing Regulation Commission Implementing Regulation(EU) No 828/2014 allows statements of “very low gluten” to food that contains no more than 100 mg of gluten/kg of food). The background for the limit is that Codex Alimentairus (2008) defined foods specially processed to reduce the gluten content to a level of 20–100 mg of gluten/kg of food as foods consisting of one or more ingredients from wheat, rye, barley, oats or their crossbred varieties that have been specially processed to reduce the gluten content to these levels. Food standards codes in Australia and New Zealand retain the 200 mg/kg limit for a “low gluten” claim (Food Standards Australia New Zealand, 2022). However, these claims do not seem to be used much by food producers in other jurisdictions.

In conclusion, the limit of 20 mg of gluten/kg gluten free food, which is based on the intake of solid foods, has been a compromise between safety and feasibility to produce and control gluten-free foods.

## 4 Reducing gluten content in beer

Malting and brewing processes during beer production have a significant impact on the protein content of beers that come primarily from barley. For example, during the malting process, where barley grains germinate, endogenous proteases partially hydrolyse gluten into more soluble peptides or larger protein fragments. In contrast to malting, mashing can partially aggregate hordeins which become insoluble precipitating in the wort or forming a layer that is typically removed during the lautering process. Although most of the protein content is removed during these processes, the final product can contain several proteins and peptides that vary greatly depending on the type of malt and brewing procedure used (Perrocheau et al., 2005). These fragments may still contain epitopes that are immunotoxic to those with coeliac disease. These can be further reduced by enzymatic proteolysis and filtration. The use of prolyl-endopeptidase (PEP) derived from *Aspergillus niger* (AN-PEP) for selective hydrolysis of gluten in beers has been described in the literature (Akeroyd et al., 2016; Decloedt et al., 2024; Di Ghionno et al., 2017; Guerdrum and Bamforth, 2012; Knorr et al., 2016; Van Landschoot, 2011). Enzymatic treatment of beer to lower gluten contents can reduce foam stability but does not have a significant impact on the beer sensory profile (Cela et al., 2023; Di Ghionno et al., 2017). This enzyme preparation, commercially available on the market as Brewers Clarex®, can, at least, partially cleave the prolamin epitopes implicated in coeliac disease. Notably, PEP was not able to efficiently cleave all proline sites in GR beers (Colgrave et al., 2017). There was high variation in detected gluten amounts across the range of commercial beers tested by liquid chromatography mass spectrometry (LC-MS); from very low levels of gluten to as much as in an untreated beer, demonstrating that PEP enzyme use is not universally optimised.

Microbial transglutaminase has also been used in attempts to detoxify gluten by cross-linking gluten peptides (Marx et al., 2008; Pasternack et al., 2006). If the cross-linked, peptide aggregates exceed a certain molecular weight, they lose their solubility and can be removed from the beer by filtration, e.g., over a layer of diatomaceous earth. A study by Taylor et al. (2015a) showed how treatment of beer with microbial transglutaminase could be a viable option for reducing levels of coeliac toxic proteins while maintaining product quality.

Regarding fining agents, brewers commonly use beer stabilisers such as silica gel or tannins to remove protein–polyphenol complexes that cause colloidal haze in beer. The application of these processing aids also leads to gluten reduction. The effectiveness in reducing gluten concentrations depends mainly on the dose of application. Silica gel proved suitable for reducing gluten levels without adversely affecting the beer's characteristics (Benítez et al., 2016; Taylor et al., 2015b). Silica gel is a synthetic porous form of silicon dioxide possessing a high affinity for proline residues. This causes the formation of large clumps that can be easily removed by filtration or precipitation. Tannins are also used in brewing as beer stabilisers. They form insoluble complexes with proteins/polypeptides in a few minutes, subsequently removed by filtration or sedimentation. Stabilisation with tannins reduced the hordein content in beer significantly. However, the high doses of tannins necessary to reduce gluten content below 20 mg of gluten/kg of food may negatively affect the sensory quality of the final beer, unlike silica gel (Taylor et al., 2015b).

## 5 Detection and quantification of gluten epitopes

One of the main problems for the quantification of gluten arises from the definition of the quantity intended to be measured (the measurand) which is total gluten content. The total gluten content is a sum parameter of a complex protein fraction of seed storage proteins. For legislative purposes gluten is defined as “a protein fraction from wheat, rye, barley, oats or their crossbred varieties and derivatives thereof, to which some persons are intolerant and that is insoluble in water and 0.5 M NaCl” (Codex Alimentarius, Codex Standard 118–1979 revised in 2008).

Total gluten content cannot be measured directly in a food matrix with current analytical capabilities, but instead, proteins, epitopes or peptides, individually or as a sum parameter, can be measured and the results converted into total gluten content. This is done to relate the quantitative data obtained to clinical relevance from which current gluten limits in legislation are derived (see section 3). This does not necessarily imply detecting pathogenic epitopes, but these techniques can target epitopes or sequences that serve as proxies and give a precise result in a wide range of processing conditions. However, in the case of gluten, section 5.1. of Codex Standard 118–1979 (Codex Alimentarius, 2008) states that the quantitative determination must be based on immunologic methods or other methods giving equal sensitivity and specificity and that “the antibody used should react with the cereal protein fractions that are toxic for persons intolerant to gluten and should not cross-react with other cereal proteins or other constituents of the foods or ingredients”. This means that the antibody used must react with epitopes from the gliadin and glutenin fractions (and the corresponding fractions from rye and barley), as these contain the proteins that cause coeliac disease and hence need to be detected and quantified. It does not specify which epitopes should be used as analytical targets, although one common interpretation is that it should be the immunotoxic epitopes.

The estimation of gluten as a sum approach of all proteins (as described by Martinez-Esteso et al., 2020, for estimation of total milk protein) seems difficult due to the diversity of proteins present. Instead, specific analytical targets must be defined. Whether they are part of T-cell epitopes or just serve as proxies, they must be robust enough to the processing conditions of the analysed food and keep a constant ratio to total gluten (Rzychon et al., 2017). This ratio is used to establish a conversion factor to derive the total gluten content in the food from the quantity of the target analyte measured, which is what regulatory bodies require. However, the diversity of proteins and protein composition that exist between species (wheat, rye, barley), between genera (*Triticum monococcum, T. dicococcum, T. aestivum*), or between cultivars adds to the complexity of measuring gluten and finding appropriate conversion factors. In addition, these measurements are done often in highly processed foods impacting on their protein composition or accessibility. Specifically, gluten proteins may be modified during processing altering the sequence or structure of the proteins. Therefore, the stability of these markers under different processing conditions and their relevant analytical behaviour must be carefully examined (Cubero-Leon et al., 2023).

Besides a clear definition of the analytical target(s), reference materials (RMs) characterised for their content can be used for calibration of different measurement procedures. These can improve the comparability of measurement results if the measurement procedures are targeting the same analytical markers which are robust enough to the processing conditions and keep a constant ratio to total gluten content, as described above. RMs were used to select wheat peptide markers representing all wheat gluten classes and a comparison of LC-MS/MS to traditional ELISA analyses and gel permeation liquid chromatography was made (Schalk et al., 2018a, Schalk et al., 2018b). The study used peptide-specific conversion factors to determine total gluten levels. Further progress has been made with regards to a wheat RM (Schall et al., 2020), rye RM (Xhaferaj et al., 2023a) and barley RM (Xhaferaj et al., 2023b) but no such materials exist for beer where the inputs and processes differ greatly.

Therefore, to improve the comparability of measurement results obtained by different measurement procedures and/or principles, additional supporting tools are needed. These include a clear definition of the analytical target(s), RMs based on these, well-documented procedures, and agreed conversion factors to transform the quantity of the analytical target into total gluten content. The different measurement procedures and principles to detect and quantify gluten, as well as their application for detecting and quantifying gluten in beer are discussed below.

### 5.1 Immunoassays

The most used methods to detect and quantify gluten are enzyme-linked immunosorbent assays (ELISA). They exploit the highly specific antibody-antigen interaction to determine gluten proteins or peptides and the amount of protein is quantified by a coloured, chemiluminescent or fluorescent signal. Polyclonal antibodies (pAbs) are produced by immunisation of mammals. Contrary to pAbs, monoclonal antibodies (mAbs) are produced in cultures of cells that derive from single immune cells (hybridoma) and they recognise a single epitope. The advantage of mAbs is that they can be produced in almost unlimited quantities with better batch to batch reproducibility and higher specificity than pAbs. However, pAbs can have a higher antibody affinity due to the recognition of multiple epitopes. Two ELISA formats, sandwich and competitive, are available for the analysis of intact gluten proteins and partially hydrolysed gluten proteins, respectively. Sandwich ELISAs require two antibody-binding sites on the antigen, because the antigen is bound between the capture Ab and the enzyme-linked detection Ab. The measured signal is directly proportional to the concentration of gluten-derived proxy peptides. In turn, competitive ELISAs only require one antibody-binding site, because enzyme-linked detection Abs compete for binding to plate-bound antigens and antigens from the sample, resulting in a signal that is inversely proportional to the concentration of gluten-derived proxy peptides.

While a wide variety of gluten- or gliadin-specific antibodies have been developed by different research groups, most commercially available ELISA test kits for gluten detection are based on the Skerritt (401.21) (Skerritt and Hill, 1990), R5 (Valdés et al., 2003), G12 and A1 (Morón et al., 2008) as well as α20 (Mitea et al., 2008) mAbs or various pAbs. Originally raised against secalins from rye, the R5 mAb mainly recognises the QQPFP and QLPFP epitopes (Valdés et al., 2003). The QQPFP epitope is present in the non-deamidated forms of five T-cell epitopes (DQ2.5-glia-γ4c, DQ2.5-glia-γ4e, DQ2.5-glia-γ5, DQ2.5-glia-ω2, DQ2.5-hor-2/DQ2.5-sec2). In gluten, these are predominantly found in ω1,2-gliadins, ω-secalins, C-hordeins and γ-75k-secalins, but also in low-molecular-weight glutenin subunits (LMW-GS), B-hordeins, γ-gliadins and α-gliadins (Lexhaller et al., 2017). The G12 and A1 mAbs were raised against the 33-mer peptide of α2-gliadin and have the highest affinity for the epitopes QPQLPY/QPQLPF and QLPYPQP/QQPFPQP, respectively (Morón et al., 2008), that mainly occur in α-gliadins, ω1,2-gliadins, ω-secalins and γ-gliadins, as well as C-hordeins, B-hordeins and γ-hordeins (Lexhaller et al., 2017). The QPQLPY epitope is part of the non-deamidated forms of three T-cell epitopes (DQ2.5-glia-α1a, DQ2.5-glia-α1b, DQ2.5-glia-α2). Recent work (Röckendorf et al., 2017) using R5 mAb, G12 mAB and the α20 mAb indicates that many coeliac disease immunotoxic sequences remain undetected, no matter which antibody is employed.

Currently, the R5 and G12/A1 mAbs are most widely used for gluten quantification. Standard testing procedures for gluten-free quality control usually employ the so-called R5 Méndez ELISA, because it is laid down in Codex Alimentarius Standard 118–1979 as a type 1 method for gluten analysis. Each commercially available ELISA test kit has specific features such as the format (sandwich or competitive), the extraction procedure, the characteristics of the Ab including its affinity towards different gluten proteins from wheat, rye and barley and the reference material used for calibration. Because all these features have an influence on the results, it is not surprising that the comparison of results from different test kits for the same sample showed a systematic bias (Bruins Slot et al., 2015; Rzychon et al., 2017; Scherf, 2017).

Detecting residual gluten in barley-based beers or other products containing partially hydrolysed gluten such as barley malt extracts, malt vinegars, soy sauces and other fermented foods by ELISA is particularly tricky, because the spectrum of gluten peptides can be extremely diverse depending on the processing conditions from the raw material to the final product (Pahlavan et al., 2016). Moreover, in beer the presence of reducing sugars can lead to glycation, a nonenzymatic modification of proteins resulting from the Maillard reaction. The addition of a sugar to a lysine residue may mask an antibody binding epitope and thus reduce the ELISA response; prevent tryptic cleavage of a protein at the glycated site, interfering with LC-MS detection; or alter the immunological response.

A common routine method used to assess regulatory compliance of such foods is the R5 competitive ELISA (Lacorn and Weiss, 2015). This test is calibrated to peptic-tryptic digests of prolamins from wheat, rye and barley flours (Gessendorfer et al., 2009). Other competitive ELISAs based on the G12/A1 mAb (Moreno et al., 2016) and the α20 mAb (Sajic et al., 2017) are also available. One multiplex competitive ELISA using five different antibody conjugates including the G12, R5 and Skerritt mAbs was developed to simultaneously detect gliadin-, deamidated gliadin-, and glutenin-specific epitopes. It recognised the different protein/peptide profiles depending on the nature of each fermented-hydrolysed food (Panda et al., 2017; Panda and Garber, 2019).

Consequently, competitive ELISAs used for gluten-free testing have some limitations, because the reference material for calibration is always a compromise and does not adequately represent the multitude of hydrolytic conditions present in different foods, depending on the process (Scherf et al., 2021). In addition, it is possible that residual gluten peptides do not contain the epitopes recognised by the mAb but do contain one of the 38 different gluten-derived T-cell epitopes that have been identified as major drivers of coeliac disease (Sollid et al., 2020).

### 5.2 Mass spectrometry

Liquid chromatography with tandem mass spectrometry (LC-MS/MS) offers potential in food analysis, despite the multitude of gluten products and the complexity of their composition, as it can simultaneously detect multiple peptides from a protein precursor enabling avoidance of protein regions or sites that might be modified during processing. LC-MS/MS can differentiate species by employing grain-specific peptide markers. There are methods developed specific to wheat (Colgrave et al., 2015), rye (Pasquali et al., 2019), barley (Colgrave et al., 2016) and oats (Dawson et al., 2018) – and multiplexed allergen detection (Gomaa and Boye, 2015; Lock, 2014; Martínez-Esteso et al., 2016).

Quantitative analysis by LC-MS/MS commonly uses the bottom-up proteomic workflow wherein proteins are solvent extracted from the food matrix before being chemically treated (reduced, alkylated) and enzymatically digested (e.g., trypsin or chymotrypsin). The peptide solutions are chromatographically separated and analysed by MS. This often involves the identification of characteristic peptide markers for a given gluten type followed by quantitative multiple reaction monitoring (MRM) analysis. LC-MRM-MS has been extensively applied to gluten detection in food and beverages (Colgrave et al., 2014, 2015, 2016; Dawson et al., 2018; Fiedler et al., 2014; Gomaa and Boye, 2015; Li et al., 2019; Lock, 2014; Pasquali et al., 2019; Schalk et al., 2018a, 2018b) including fermented products (Colgrave et al., 2012; Li et al., 2018) or after treatment with enzymes that aim to remove gluten (Colgrave et al., 2014; Fiedler et al., 2018, 2019; Spada et al., 2020; Tanner et al., 2013).

During processing, including malting and brewing, gluten is hydrolysed to yield peptides or larger protein fragments. These fragments may result in false negative results by ELISA if the fragments do not contain the antigenic epitopes recognised by the ELISA antibody. However, they may still contain immunogenic epitopes that can elicit a response in those with coeliac disease. LC-MS/MS analysis of 12 commercial beers that were marketed as “reduced gluten” or “gluten free” (Colgrave et al., 2017) revealed gluten fragments across three different size ranges. Notably, in several GR beers, the gluten peptide markers were detected at similar levels to that found in control beers, that had not been processed to reduce gluten. Additionally, some gluten proteins were noted to be more resistant to digestion by the PEP enzyme than others. Using MS, the gluten-reduction strategy, i.e., use of the PEP enzyme, can be revealed by assessing protein hydrolysis rates at proline residues (Colgrave et al., 2017).

Despite the benefits of LC-MS/MS, several constraints exist as it pertains to the proteomic workflow, including: (1) the variability in extraction efficiency that can result from food processing (thermal/pressure/enzyme treatments); and (2) the requirement for enzymatic digestion for peptide liberation noting that trypsin digests do not yield peptides across all gluten classes, thus necessitating other enzymes like chymotrypsin to be used in parallel. Furthermore, targeted methods such as LC-MRM-MS share a common drawback with immunochemical methods in that the analytical targets must be defined *a priori*. This can be circumvented by undertaking untargeted MS analyses but the variability of raw materials and brewing technology, combined with instrument differences render standardisation of measurement across laboratories difficult to achieve. Additionally, limitations remain in the incomplete nature of public databases that hinder the accurate and complete identifications in proteomic analyses. In fermented products wherein extensive hydrolysis is present/expected, this requires the use of “no enzyme” searches, which can negatively impact the confidence of peptide/protein identifications. From a quantitative viewpoint, one critical consideration is the conversion of MS reporting metrics (i.e., peptide peak areas) to total gluten levels as needed by regulatory bodies requiring appropriate and well characterised reference materials RMs.

## 6 Interpretation of gluten measurement and risk assessment

While advances have been made in detection and analysis of gluten peptides in beer, several challenges remain for the interpretation of gluten measurement for proper risk assessments. What matters for coeliac patients are which immunotoxic gluten peptides face their gut immune system at what concentrations. Any risk assessments should ideally be based on this fact. However, in real life today no risk assessment of gluten toxicity fully embraces this scenario. An obvious limitation is the current lack of knowledge of all immunotoxic sequences. The reactivity of only 50 % of gluten-specific T cells of coeliac disease patients can be accounted for by peptides representing the currently available list of 38 nine-mer sequences (Sollid et al., 2020). More T-cell epitopes are to be identified. Current clinical data is based on gluten intake and analytical measurements must report total gluten content (see section 5). These consumption safety estimates are based on solid food not considering that hydrolysed peptides in liquids, such as the case of beer, may be handled differently in vivo.

In addition to the issue of not knowing all immunotoxic sequences, there are several challenges related to the interpretation of the detection of sequences. The limitations of immunoassays are discussed above. While the antibodies used may or may not directly detect sequences representing T-cell epitopes, no antibody-based assay covers all gluten epitopes. While MS identifies peptide sequences, the detection is often not quantitative – and amount does matter. Further, MS may identify peptides which do not harbour any of the defined nine-mer core regions of T-cell epitopes, but which can have striking similarities with some of the sequences. Whether such peptides are truly harmful to coeliac disease patients are impossible to predict with the tools we have available today.

A much-used tool to assess if there are coeliac disease associated protein structures in foods is allergenOnline.org. AllergenOnline.org is a screening tool developed to assess if there are coeliac disease associated protein structures in novel foods (Goodman et al., 2016; Amnuaycheewa et al., 2022) and to identify proteins that may require additional tests. It contains native and deamidated peptides, 1013 peptides (in the 2018 version) with average length 16 ± 4 amino acids that are reported to stimulate CD4^+^ T cells with a proliferation with greater than a 2-fold stimulatory index or release of IFN-gamma, or have been shown to give toxic reactions in the intestines of patients with coeliac disease (9 peptides) (Amnuaycheewa et al., 2022). The current list of 1041 peptides is derived from 75 publications (allergenOnline.org download March 2024). The criteria for defining T-cell stimulation here is much less stringent than the criteria defined by Sollid et al. (2012). Of note is that the original reports referred in the database often have been done on polyclonal T-cell responses with no insights into the HLA-restriction elements used by the T cells. Thus, it is unclear whether all the entries of this database represent peptides that are stimulatory to HLA-DQ2.5, HLA-DQ2.2 or HLA-DQ8 restricted T cells of coeliac disease patients. Another issue is that while allergenOnline.org specifies on the website that “*Proteins that do not contain an exact peptide match to those identified in this database are unlikely to induce symptoms in those with coeliac disease*” some experts in the analytical community, suggest that peptide sequences only having partial matches in AllergenOnline.org may pose a risk for patients with coeliac disease (Fiedler et al., 2019).

## 7 Challenges ahead

Based on current knowledge it is difficult to assess whether barley-based gluten-free beer is safe for consumers with coeliac disease.

As CD4^+^ T cells are considered the drivers of immunopathology of coeliac disease, a comprehensive definition of the epitopes recognised by these gluten-specific CD4^+^ T cells will be essential. Not all T-cell epitopes have been defined as yet, and further identification of T-cell epitopes in barley will be important for proper assessment of beers. Assessing safety of peptides found in gluten-reduced beers just by homology with published gluten T-cell epitopes, as some current sequence databases do, may be misleading (see section 6).

There are analytical challenges to control and monitor the production of gluten-free beer. While there is a controversy whether antibodies and immunoassays give reliable quantitative assessment of gluten content, it remains a fact that many antibody assays target sequences which are not T-cell epitopes, and no antibody assay targets all immunotoxic gluten T-cell epitopes or total gluten. The antibody-based immunoassays provide useful information though, not least because they provide quantitative assessments. The main problem is to relate the information obtained from such assays to the in vivo toxicity of beer. Ideally, recommendations as to whether beers are safe to coeliac disease patients will require some sort of in vivo toxicity assessment. Clinical feeding studies with small-bowel histology is considered the best approach to establish adverse clinical effects. Current gluten limits were estimated from a clinical feeding study with small-bowel biopsies using whole gluten (Catassi et al., 2007). In beer, there are short peptides which likely are handled by the gut digestive system different from whole gluten in a food matrix. It will be important to conduct feeding studies of coeliac disease patients with analytically characterised protein/peptide extracts of beer.

In summary, analysts are at present aiming to be very accurate in quantifying gluten in gluten free beer, yet there is uncertainty about the clinical relevance of the degraded proteins. We recommend future progress will require.

1. In vivo toxicity assessments of the degraded proteins in coeliac disease patients;
2. Development of standardised gluten-free production strategies to minimise variability in resulting gluten fragments and guidance aimed at producers and authorities on how to control the outcome;
3. Development of appropriate RMs and calibrators based on (1) and (2).

The analytical community is keen to collaborate with relevant input to the above steps which will enable subsequent focus on identifying suitable targets for gluten quantification in ‘gluten-free’ beer. The authors trust that this paper is seen as a first step in such collaboration.

## CRediT authorship contribution statement

**Elena Cubero-Leon:** Writing – review & editing, Writing – original draft, Supervision, Conceptualization. **Charlotte B. Madsen:** Writing – review & editing, Writing – original draft, Conceptualization. **Katharina A. Scherf:** Writing – review & editing, Writing – original draft, Conceptualization. **Michelle L. Colgrave:** Writing – review & editing, Writing – original draft, Conceptualization. **Jørgen V. Nørgaard:** Writing – review & editing, Writing – original draft, Conceptualization. **Minna Anthoni:** Writing – review & editing, Writing – original draft, Conceptualization. **Katerina Rizou:** Writing – review & editing, Writing – original draft, Conceptualization. **Michael J. Walker:** Writing – review & editing, Writing – original draft, Conceptualization. **Ludvig M. Sollid:** Writing – review & editing, Writing – original draft, Conceptualization.

## Declaration of competing interest

The authors declare that they have no known competing financial interests or personal relationships that could have appeared to influence the work reported in this paper.

## Data Availability

No data was used for the research described in the article.

## ABBREVIATIONS:

ELISA: enzyme-linked immunosorbent assay
GR: Gluten-removed
HLA: Human leukocyte antigen
LC-MS: Liquid chromatography-mass spectrometry
LC-MS/MS: Liquid chromatography with tandem mass spectrometry
mAbs: monoclonal antibodies
MHC: Major histocompatibility complex
MRM: multiple reaction monitoring
MS: mass spectrometry
NOAEL: No Observed Adverse Effect Level
pAbs: polyclonal antibodies
PEP: Propyl-endopeptidase
RM: reference material
TG2: transglutaminase 2
WHO: World Health Organisation
